# Cyclic Progesterone Therapy in Androgenic Polycystic Ovary Syndrome (PCOS)—A 6-Month Pilot Study of a Single Woman’s Experience Changes

**DOI:** 10.3390/medicina57101024

**Published:** 2021-09-26

**Authors:** Sonia Shirin, Faye Murray, Azita Goshtasebi, Dharani Kalidasan, Jerilynn C. Prior

**Affiliations:** 1 Centre for Menstrual Cycle and Ovulation Research, Endocrinology, University of British Columbia, Vancouver, BC V5Z 1M9, Canada; sonia.shirin@ubc.ca (S.S.); fvmurray@gmail.com (F.M.); azita.goshtasebi@ubc.ca (A.G.); dhani.kalidasan@ubc.ca (D.K.); 2 British Columbia Women’s Health Research Institute, Vancouver, BC V6H 3N1, Canada; 3 Division of Endocrinology, Department of Medicine, University of British Columbia, Room 4111, 2775 Laurel Street, Vancouver, BC V5Z 1M9, Canada; 4 School of Population and Public Health, University of British Columbia, Vancouver, BC V6T 1Z3, Canada

**Keywords:** androgenic PCOS, cyclic oral micronized progesterone therapy, menstrual cycle diary, fluid retention, breast tenderness

## Abstract

*Background and Objectives:* Women with androgenic Polycystic Ovary Syndrome (PCOS) have increased endometrial cancer risk that cyclic progesterone will prevent; it may also reverse PCOS’s neuroendocrine origins. This pilot study’s purpose was to document 6-month *experience changes* in a woman with PCOS taking cyclic progesterone therapy because she was intolerant of combined hormonal contraceptive therapy, the current PCOS standard of care. A 31-year-old normal-weight woman with PCOS had heavy flow, irregular cycles, and was combined hormonal contraceptives-intolerant. She was prescribed cyclic oral micronized progesterone (OMP) (300 mg/*h.s.* cycle days 14–27). She kept Menstrual Cycle Diary© (Diary) records, starting with the 1st treatment cycle for six cycles; she was on no other therapy. Statistical analysis *a priori* hypothesized progesterone decreases high estradiol (E2) experiences (flow, cervical mucus, fluid retention, front-of-the-breast tenderness and anxiety); analysis focused on these. Our objectives: (1) changes from cycles 1 to 6 in E2-related experiences; and (2) follicular phase E2-related changes from cycle 1 (no therapy) to cycles 3 and 6. *Materials and Methods:* Data from consecutive Diaries were entered into an SPSS database and analyzed by Wilcoxon Signed Rank Test (Objective #1) within-person whole cycle ordinal data, and (Objective #2 follicular phase) repeated measures ANOVA. *Results:* Cyclic OMP was associated with regular, shorter cycles (±SD) (28.2 ± 0.8 days). Comparison of cycles 1–6 showed decreased fluid retention (*p* = 0.001), breast tenderness (*p* = 0.002), and cervical mucus (*p* = 0.048); there were no changes in flow or anxiety. Fluid retention in the follicular phase also significantly decreased over time (F (1.2, 14.7) = 6.7, *p* = 0.017). *Conclusions:* Pilot daily Diary data suggest women with PCOS have improved everyday experiences on cyclic progesterone therapy. Larger prospective studies with more objective outcomes and randomized controlled trials of this innovative PCOS therapy are needed.

## 1. Introduction

Androgenic Polycystic Ovary syndrome (PCOS) is a common problem for women of reproductive age [1]. In a meta-analysis of random population-based studies, PCOS occurs in 10% (95% Confidence Interval (95%CI) 7–14%) of all women [2]. Women living with PCOS face many health problems, some of which have sociocultural dimensions, such as difficulty in losing weight [3], excess facial hair, acne, [4] alopecia, and infertility [3], with resulting increases in depression and anxiety [5]. Women with PCOS almost always experience oligomenorrhea [6] as well as being at increased risk for endometrial cancer [7]. Combined hormonal contraceptives (CHC) are the current standard of care for women with PCOS [1]. Although CHC shortens and causes regular menstrual cycles, while somewhat improving acne and hirsutism [8], all except acne-related benefits largely disappear after six months off CHC [9].

A number of causes for PCOS are postulated, including androgen exposure *in utero*, genetic risks, and inflammation [10]; however, increasing evidence suggests that PCOS is characterized by a central dysregulation of the hypothalamic–pituitary–ovarian axis with rapid pulsing of gonadotrophin releasing hormone [11,12]. This results in rapidly pulsing luteinizing hormone (LH), which stimulates ovarian androgen production and prevents normal ovulation. Cyclic progesterone therapy may improve these fundamental PCOS changes [13,14]; progesterone physiologically slows LH pulsatility at the menstrual midcycle [15]. 

If our hypothesis about the hypothalamic/neuroendocrine origin of PCOS is correct, cyclic oral micronized progesterone therapy (Cyclic OMP; 300 mg at *h.s*. for 14 days per cycle) could provide treatment for women with PCOS who are unable to tolerate CHC treatment, correct the neuroendocrine *origins* of PCOS, as well as providing regular cycles and decreasing the risks for endometrial cancer [7]. Women with PCOS are exposed to tonically elevated E2 levels without the normal decreases during flow [16,17]. In a randomized placebo-controlled crossover trial of daily treatment with 300 mg of oral micronized progesterone for premenstrual symptoms, it significantly decreased anxiety, depression, breast tenderness and fluid retention in premenopausal women [18]. Short cycle Cyclic OMP (for 7 or 10 days) has been documented to decrease LH and/or testosterone (T) [19,20]. However, there are no published person-level data on the *experiences* of women with PCOS *while they are taking Cyclic OMP*. Our purpose was to conduct a prospective pilot study systematically documenting the 6-month experience changes on Cyclic OMP therapy for one woman with PCOS. 

## 2. Materials and Methods

A 31-year old woman (BMI 20.1), with previous heavy flow and slightly irregular ~35-day long cycles, was unable to tolerate CHC. She was prescribed Cyclic OMP (300 mg/*h.s.* on cycle days 14–27) [12]. Beginning at the start of the cycle in which she began Cyclic OMP, she began recording a Menstrual Cycle Diary© (Diary) [21], a 19-item tool, with each variable ordinal-scored on a 0 to 4 scale; she took no other therapy. 

Given our hypothesis that progesterone would decrease the experiences often related to high estradiol levels (heavy flow, fluid retention, cervical mucus, front-of-the-breast tenderness and anxiety), we had two objectives: (1) Document experience changes across the whole cycle in selected, high-estrogen-related experiences between cycles 1 and 6 by Wilcoxon Signed Rank Test (for within-person ordinal data); (2) Compare follicular phase Diary changes by repeated measures ANOVA from cycle 1 (no Rx until cycle day 14) with cycles 3 and 6, during which she was taking Cyclic OMP. All Diary data were entered into an SPSS (IBM SPSS 24, New York, NY, United States) database. The participant provided signed and witnessed informed consent.

## 3. Results

This highly symptomatic young woman with androgenic PCOS was treated for six cycles with “luteal phase replacement” doses and durations of cyclic oral micronized progesterone. She spontaneously reported that while taking Cyclic OMP, she noted important improvements in aching joints, disturbed sleep and gastrointestinal symptoms. 

**Figure 1:**
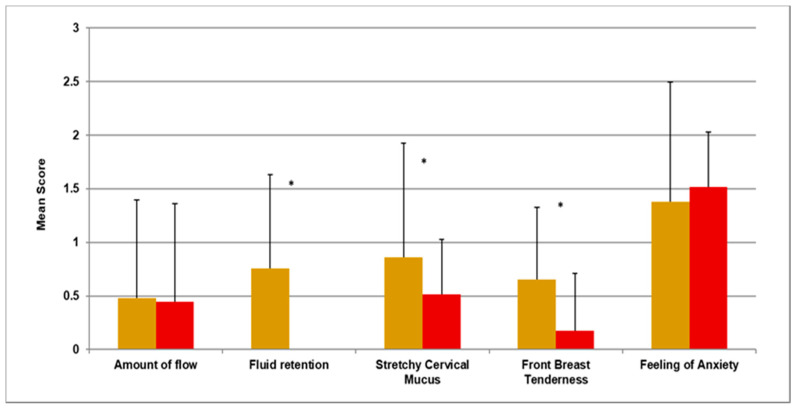
Cycle 1 (gold) versus 6 (red) using the Wilcoxon Paired Signed Ranks Test. * noted above data indicates a significant change of *p* ≤ 0.05.

In our first objective related to whole cycle analysis, Cyclic OMP was associated with shorter cycles that were very regular, with lengths of 28.2 ± standard deviation 0.8 days. Fluid retention (*p* = 0.001), stretchy cervical mucus (*p* = 0.048), and front-of-the-breast tenderness (*p* = 0.002) all significantly decreased. The amount of flow and feelings of anxiety were unchanged (Figure 1). 

In our second objective with the follicular phase only repeated measures analysis, decreased fluid retention (F (1.2, 14.7) = 6.7, *p* = 0.017) was the only significant difference between the untreated baseline and cycles 3 and 6. She reported no adverse experiences during this study. She requested a renewed and ongoing Cyclic OMP prescription from her physician.

## 4. Discussion

This pilot prospective, 6-month study within one woman with androgenic PCOS during Cyclic Progesterone Therapy showed predictable withdrawal flow and beneficial changes in cycle length and regularity. In addition, she experienced significantly improved fluid retention, front-of-the-breast tenderness and cervical mucus secretions. These novel data are without any comparison in the literature. The closest published data are the questionnaire data obtained during daily oral micronized progesterone therapy (300 mg/day) in premenopausal women of a similar age being treated for premenstrual symptoms [18]. 

Although progesterone is commonly used for oligo-amenorrhea assessment [22] (the so called “progestin challenge” test) or for treatment [23], available published data suggest that cyclic progesterone therapy may be beneficial in PCOS. Two PCOS short-cycle (7 and 10 day) progesterone studies documented decreased serum LH and T levels [19,20]. Vaginal progesterone therapy was also associated with decreased LH levels, and LH pulse rates slowed to normal [24]. All studies reporting flow also documented, as did this study, that menstrual bleeding began within a day or two of stopping the progesterone.

Tonically elevated estradiol/estrone levels [16] and lower progesterone levels likely cause the increased endometrial cancer rates reported in PCOS [7]. A randomized, double blind, crossover trial with OMP 300 mg/day among premenopausal women with premenstrual symptoms showed significantly reduced fluid retention, breast discomfort, anxiety and depression [18]. 

Limitations of this pilot study are that it includes only self-reported data, is unblinded, and in a single woman. The strengths of this study are that we have collected standardized Diary data [21], using which we have previously published one-year menstrual cycle changes in negative moods, fluid retention and interest in sex, respectively, in normally ovulatory women [25,26,27]. It is also a strength that we gave Cyclic OMP over six months, which is much longer than the one- or two-cycle progesterone studies in women with PCOS reported in the literature [19,20]; we also gave it for an ideal 14-day cycle rather than 7–10 days, as has been reported. Finally, we appropriately analyzed these ordinal data within one woman over time using non-parametric statistics. 

## 5. Conclusions

Although open-label and in a single woman, these novel prospective data suggest that cyclic oral micronized progesterone (Cyclic OMP), given as a sole therapy, is associated with beneficial effects in experiences in one woman with androgenic PCOS. Prospective, larger, within-woman PCOS studies are required in which changes in quality of life and objective anthropomorphic, hormonal, and metabolic data are documented before and at the end of Cyclic OMP therapy. Randomized, controlled trials also remain necessary. 

## Data Availability

Anonymized primary data are available for well-described objectives to the senior author on request from qualified researchers.
